# Colorectal Cancer-Infiltrating Regulatory T Cells: Functional Heterogeneity, Metabolic Adaptation, and Therapeutic Targeting

**DOI:** 10.3389/fimmu.2022.903564

**Published:** 2022-07-08

**Authors:** Sonia Aristin Revilla, Onno Kranenburg, Paul J. Coffer

**Affiliations:** ^1^ Center Molecular Medicine, University Medical Center Utrecht, Utrecht, Netherlands; ^2^ Regenerative Medicine Center, University Medical Center Utrecht, Utrecht, Netherlands; ^3^ Laboratory Translational Oncology, University Medical Center Utrecht, Utrecht, Netherlands

**Keywords:** colorectal cancer, CRC, T regulatory cells, Treg, tumor microenvironment, immunotherapy, immunometabolism, tumor infiltrating cells

## Abstract

Colorectal cancer (CRC) is a heterogeneous disease with one of the highest rates of incidence and mortality among cancers worldwide. Understanding the CRC tumor microenvironment (TME) is essential to improve diagnosis and treatment. Within the CRC TME, tumor-infiltrating lymphocytes (TILs) consist of a heterogeneous mixture of adaptive immune cells composed of mainly anti-tumor effector T cells (CD4+ and CD8+ subpopulations), and suppressive regulatory CD4+ T (Treg) cells. The balance between these two populations is critical in anti-tumor immunity. In general, while tumor antigen-specific T cell responses are observed, tumor clearance frequently does not occur. Treg cells are considered to play an important role in tumor immune escape by hampering effective anti-tumor immune responses. Therefore, CRC-tumors with increased numbers of Treg cells have been associated with promoting tumor development, immunotherapy failure, and a poorer prognosis. Enrichment of Treg cells in CRC can have multiple causes including their differentiation, recruitment, and preferential transcriptional and metabolic adaptation to the TME. Targeting tumor-associated Treg cell may be an effective addition to current immunotherapy approaches. Strategies for depleting Treg cells, such as low-dose cyclophosphamide treatment, or targeting one or more checkpoint receptors such as CTLA-4 with PD-1 with monoclonal antibodies, have been explored. These have resulted in activation of anti-tumor immune responses in CRC-patients. Overall, it seems likely that CRC-associated Treg cells play an important role in determining the success of such therapeutic approaches. Here, we review our understanding of the role of Treg cells in CRC, the possible mechanisms that support their homeostasis in the tumor microenvironment, and current approaches for manipulating Treg cells function in cancer.

## Introduction

Colorectal cancer (CRC) is the third most diagnosed cancer worldwide, and the second main cause of cancer-related death (1). The main causes of high mortality are late diagnosis and high relapse after treatment, with 70% of patients only being diagnosed when the tumor has metastasized to distant organs such as liver (20%-70%) and lung (10%-20%) (2, 3). CRC patients are a heterogeneous group, where differences at the molecular and genetic level influence clinical outcomes and response to therapy (4, 5). Seventy percent of CRC is of mostly sporadic origin, while 20-30% of cases are familial. From these, approximately one-third are caused by highly penetrant inherited mutations that have been well characterized, so-called hereditary CRC. The etiology of the remaining familial cases is not completely understood but is often associated with lower-penetrance susceptibility genes and specific polymorphisms regulated by environmental, or other genetic inherited factors, that when co-inherited increase cancer risk (6–8). Additionally, intestinal inflammatory responses related to inflammatory bowel diseases (IBDs) can result in the development of CRC (9). Prolonged and severe inflammation often correlates with increased cancer risk (10). Colorectal polyps or adenomas arise from the epithelial cells lining the colon and are considered precursors to tumor development. During the initiation of CRC, epithelial cells acquire genetic and epigenetic modifications increasing the risk of malignant transformation and conferring a selective advantage. These can increase in size and the degree of dysplasia eventually leading to the development of dysplastic adenomas that subsequently evolve to adenoma-carcinoma (4, 5). The immune response can recognize and target tumor-specific and tumor-associated neo-antigens thereby arresting CRC development. Here, CD8+ cytotoxic T lymphocytes (CTLs) play an essential role, with the cooperation of CD4+ helper T (Th) cells. However, CRC tumors can manipulate the tumor microenvironment (TME), promoting cancer outgrowth through regulation of immune infiltration, and generating an immunosuppressive and inflammatory environment. In this way, the TME plays a key role in modulating the plasticity of tumor cells and providing immune escape mechanisms (11–14).

## The CRC Tumor Microenvironment and Tumor-Infiltrating Lymphocytes

The TME includes malignant tumor cells together with immune cells, stromal cells, endothelial cells, extracellular matrix (ECM), cytokines, chemokines, and other soluble mediators (14–17). Cancer development in general, and CRC progression in particular, are linked with the complex role that the immune response plays in the early stages of tumor development (15, 18–20). The immune system has a multi-faceted role in CRC development (15) and has a substantial impact on patient outcome (18). Tumor-infiltrating immune cells (TIICs) include adaptive immune cells (T cells and B cells), natural killer (NK) cells, macrophages, and additional innate cells such as myeloid-derived suppressor cells (MDSCs) (14, 21, 22). TIICs directly participate in the generation and maintenance of an actively suppressive anti-tumor immune response supporting CRC progression (23). MDSCs secrete cytokines and express immunosuppressive molecules at their surface that can inhibit T cells, B cells, and NK cells while promoting Treg cells and tumor-associated macrophages (TAMs) (24, 25). The most enriched population of TIICs are tumor-infiltrating lymphocytes (TILs) that promote inflammation and are often used to evaluate disease prognosis (26, 27). TILs are a heterogeneous mixture of lymphocytes composed mainly of anti-tumor effector T cells (CD4+ and CD8+ subpopulations) and immunosuppressive Treg cells (27, 28). The balance between these two populations is critical in anti-tumor immunity. In general, while tumor antigen-specific T cell responses are observed, tumor clearance frequently does not occur, highlighting the important role of immunosuppressive Treg cells (29, 30). Since tumors can directly shape TIICs populations and function, this interplay provides the capacity to escape immunosurveillance (15, 18). TILs are also associated with the molecular classification of CRC tumors, and with patient prognosis and immunotherapy responses (21, 23, 31). CRC-tumors composed of mainly anti-tumor T cells, particularly type 1 helper T cells (Th1) and cytotoxic CD8+ T cells, correlate with favorable prognosis and survival (32, 33). In contrast, increased accumulation of Treg cells is generally associated with CRC progression and metastasis, immunotherapy failure, and a poorer prognosis, although this correlation is not definitive (34–40). Understanding the dynamics between CRC tumors and immune cells in the TME is essential to improve both diagnosis and treatment.

## FOXP3+ Regulatory T Cells

FOXP3+ regulatory T cells, hereafter termed Treg cells, are essential in the maintenance of immunological homeostasis and self-tolerance (41–43). They can have broad suppressive activity, secreting immunomodulatory cytokines and cytolytic molecules allowing them to regulate immune responses (44). Treg cells have been well-studied due to their capacity to regulate the function of a wide variety immune cells including lymphocytes, dendritic cells (DCs), and macrophages (42). The depletion or reduction of Treg cells is associated with beneficial anti-tumor immune responses and eradication of microbes during chronic infections (45, 46). The concept of Treg cells as suppressor T cells was first proposed in the 1970s (47–49) and was later further defined as a subpopulation of approximately 10% of peripheral CD4+ T-cells expressing the IL-2 receptor alpha-chain (CD25) (50). However, the use of CD25 to identify Treg cells is problematic since it is a general marker of activated T-cells (51, 52). Subsequently, in a breakthrough for the field, the forkhead box P3 transcription factor (Foxp3) was found to be an additional marker of Treg cell ontogeny (53–57). Subsequent studies in both mice and humans identified FOXP3 is a master regulator of both Treg cell development and function (58, 59). Naïve CD4+ T cells can transiently upregulate CD25 and FOXP3 expression upon activation (60) and human Treg cells are usually characterized by the expression of FOXP3, CD25, and the low expression of the IL-7 receptor alpha chain, CD127 (61).

In the thymus, tTreg cell differentiation is driven by strong T cell receptor (TCR)-mediated recognition of self-antigens (62, 63). In secondary lymphoid organs, peripheral Treg cells (pTreg) can differentiate from naïve or effector CD4+ T cells through TCR stimulation in the presence of TGF-β and IL-2, inducing FOXP3 expression (63–65). The pTreg cell TCR repertoire recognizes foreign-antigens, as opposed to tTreg that are biased towards self-recognition (66, 67). Furthermore, the expression of transcription factor Helios and the cell-surface glycoprotein neuropilin-1 (Nrp1) can be used to distinguish tTreg that highly express both factors from pTreg that poorly express them. However, pTreg can also upregulate Helios and Nrp1 under inflammatory conditions, or in response to certain activation signals such as IL-2 stimulation (68–70).

FOXP3-expressing CD4+ T cells can be further subdivided into two main subsets: central Treg cells (cTreg cells) and effector Treg cells (eTreg cells). However, in humans, activated Teff cells can transiently express FOXP3 and secrete pro-inflammatory cytokines (IL-17, IL-2, and IFNγ), however they are considered to be non-suppressive (71, 72). cTreg cells are recent thymic emigrants that have not yet been activated and exhibit a naïve phenotype (CD45RA+ FOXP3lo) with low suppressive activity. They are enriched in lymphoid tissues where they express lymphoid-tissue homing molecules including CD62Lhigh, CD44low and CCR7, and are dependent on IL-2 for maintaining their quiescent state (73). In secondary lymphoid organs, cTreg cells undergo further differentiation upon TCR engagement to become eTreg cells (CD45RA- FOXP3hi). These cells proliferate, further and develop into highly suppressive cells, upregulating activation markers, immunosuppressive cytokines, chemokines, and their receptors. They subsequently migrate to non-lymphoid tissues, downregulate lymphoid-tissue homing molecules (CD62Llow, CD44high) and acquire a tissue-specific transcriptional signature associated with their role in each location (63, 74, 75). eTreg cell suppressive function correlates with co-expression of FOXP3 and lineage-specific transcription factors common the Teff cells population (**Table 1**).

**Table 1:** Treg cells subtypes.

			Characterize by	Localization	Function
**Ontogeny**	**tTreg cells**		CD45RA+ FOXP3^lo^ Helios^hi^ Nrp1 ^hi^	Thymus	tTreg cell TCR repertoire recognizes self-antigens
	**pTreg cells**		CD45RA- FOXP3^hi^ Helios^lo/-^ Nrp1 ^lo/-^	Secondary lymphoid organs	pTreg cell TCR repertoire recognizes tissue specific and foreign antigens
**Function**	**cTreg cells**		CD45RA+ FOXP3^lo^ CD25^lo^ CD62L^hi^ CD44^lo^ CCR7^hi^, secondary lymphoid tissue homing molecule	Leave the thymus to lymphoid tissues, enriched in secondary lymphoid organs	Naïve phenotype with low suppressive activity Control autoimmune reactions and induce transplant tolerance
	**eTreg cells**		CD45RA- FOXP3^hi^ CD25^hi^ CD62L^lo^ CD44^hi^ CCR7^lo^ Activation molecules: ICOS and OX-40 Inhibitory molecules: CTLA, PD-1, TIGIT and LAG-3 Effector molecules: IL-10, TGF-β, IL-35	Originated in secondary lymphoid organs and migrate to non-lymphoid tissues	Highly suppressive cells Maintain immune homeostasis OX-40+ and ICOS+ Treg cell: secrete high levels of suppressive cytokines such as IL-10 CTLA+, PD-1 and TIGIT+ Treg cell: impair dendritic cell function preventing Teff activation
	**eTreg cell subtypes**	**Th1-like Treg cells**	T-bet+ IFNγ+ CXCR3, migration to inflamed tissue mediated by Th1 cells	Inflammatory loci in non-lymphoid tissues	Inflammatory autoimmunity Secrete IFNγ and suppress Th1 and Th17 pro-inflammatory immune response
		**Th2-like Treg cells**	Gata3+ IRF4+ IL4+ CCR8, recruitment to inflamed tissue mediated by Th2 cells	Inflammatory loci in non-lymphoid tissues	Secrete IL-4 and IL-13 and suppress Th2- mediated response
		**Th17-like Treg cells**	RORγt+ IL-17+ CCR6, recruitment to inflamed tissue mediated by Th17 cells	Inflammatory loci in non-lymphoid tissues	Oral tolerance Mucosal immunity, maintain homeostasis and tolerance to commensal microbiota Secrete anti-inflammatory IL-10 to inhibit pathogenic Th17 cell responses

Histone post-translational modifications and DNA methylation patterns are intimately associated with the differentiation and function of Treg cells (76–78). FOXP3 transcription is epigenetically controlled through regulation of its promoter and several intronic enhancers, termed conserved non-coding DNA sequences (CNS) 0–3 (77, 79). The demethylation status of CNS1 and CNS2 is regulated by TGF-β and IL-2 and associated with the stable expression of Foxp3 (80–82). TET1/2 methylcytosine dioxygenases demethylate CNS1/2 thereby stabilizing *Foxp3* expression and other Treg cell signature genes including *Cd25*, *Nrp1*, and *Il1rl1* (82). Mice with CNS1‐deficiencies can generate tTreg cells but not pTreg cells, leading to a deficiency of colonic Treg cells, development of aggressive inflammation in the mucosa, and spontaneous colitis (77, 83, 84). For tTreg cell stability, Foxp3 expression alone is insufficient and additionally requires genome-wide CpG hypomethylation driven by TCR stimulation (85).

The mechanisms by which Treg cells modulate immune suppression have been the subject of many studies (43, 44, 86). In general, Treg cells exert their immunosuppressive function through three main mechanisms. Firstly, Treg cells repress the immune response of effector immune cells by secreting immunomodulatory cytokines and cytotoxic molecules. Secondly, Treg cells express immune checkpoint receptors by which they interact and suppress Teff cells or antigen-presenting cells. Furthermore, Treg cells interfere with the metabolism of effector cells thereby affecting their function.

## Tissue-Resident Intestinal Treg Cells

Over the last decade, it has become clear that Treg cells undergo tissue-specific adaptation in non-lymphoid tissues and acquire context-dependent tissue-specific gene signatures (75, 87, 88). As described above, cTreg cells express lymphoid tissue homing molecules involved in trafficking to secondary lymphoid organs. In these secondary organs, tissue-specific self-antigens or microbial antigens recognized by cTreg cells lead to their differentiation to eTreg cells, upregulating activation markers (CD44hi), effector molecules (CTLA4, GZMB, KLRG1), chemokines and their receptors (CCR4), and immunosuppressive cytokines (IL-10) (75, 89). Upon TCR stimulation, the upregulation of homing receptors, including chemokine receptors and adhesion molecules, further directs the migration and localization of the eTreg cells to non-lymphoid peripheral sites in response to specific stimuli (89).

Tissue-resident eTreg cells are found in almost all peripheral tissues where they adapt to environmental cues and exhibit a tissue-specific transcriptional adaptation associated with their function (88, 90). They are specialized in controlling peripheral immune homeostasis by acquiring a combination of homing receptors, transcription factors, immune-regulatory mechanisms, and a specific TCR repertoire (74). eTreg cells not only dampen immune responses but also promote the regeneration and repair of injured tissue or stimulate stem cell differentiation (91, 92). In the intestinal tract, the majority of the immunoregulatory processes occur in the mucosal lamina propria (LP). Among the total CD4+ T-cell population in the LP, 10-15% in the small intestine and 25-35% in the large intestine are Treg cells. To maintain intestinal immune homeostasis, Treg cells control immune responses against innocuous food and microbial antigens (75, 93). Colonic pTreg cells are mainly directed towards microbial antigens, as highlighted in studies using germ-free mice, where a reduced number of colonic Treg cells was observed compared to specific pathogen-free (SPF) mice (94). Some colonic eTreg cells originate from tTreg cells and are characterized by expression of the transcription factors Gata3, Helios, and Nrp1 (95). However, most eTreg cells result from the conversion of CD4+Foxp3− T cells to pTreg cells expressing the nuclear hormone receptor RORγt, with low Helios or Nrp1 expression (96). Control of Foxp3 expression in pTregs generated in the digestive-system lymphoid-tissue has been attributed to regulation of CNS1 (77). CNS1 contains binding sites for several transcription factors including NFAT, Smad, and retinoic acid receptor (RAR) and retinoid X receptor (RXR) whose binding promotes *Foxp3* expression. TCR stimulation activates NFAT, while TGF-β signaling activates Smad3 whose association with CNS1 contributes to histone acetylation and enhancer activation. This synergistic binding of NFAT and Smad to CNS1 is essential for *Foxp3* expression (83).

Transcriptional and functional analysis of both murine and human colonic Treg cells has defined two specialized subsets based on the expression of transcription factors and cell surface markers. One population, the FOXP3+ GATA3+ Helios+ Nrp1+ Treg cells are similar to so-called Th2-like eTreg cells. GATA3 is a canonical Th2 transcription factor with a fundamental role in controlling Treg cell fate and accumulation in tissues during inflammation. It is expressed in Treg cells homing to barrier sites such as skin and intestine, constituting one-third of the colonic Treg cells (97). Most of the GATA3+ Treg cells also express IL-1RL1 (also known as ST2), whose expression is GATA3-dependent. ST2 binds to tissue-danger signal IL-33, which is elevated in the colon after inflammation-driven tissue damage. The IL-33-ST2 pathway is crucial for Treg cell homing and accumulation in the intestine, along with high expression of the intestinal-homing receptors CCR9 and α4β7. In a positive feedback loop, IL-33 induces the recruitment of GATA3 to the *il1r1* enhancer and *foxp3* promoter, resulting in increased FOXP3 and ST2 expression establishing the eTreg cell transcriptional program. GATA3+ ST2+ Treg cells also secrete amphiregulin, a tissue-remodeling factor that mediates tissue-regeneration, and IL-10, a cytokine that supports intestinal stem cell renewal, restraining their proliferation and aberrant differentiation (98–100). In this way, Treg cell accumulation limits tissue damage by a rapid adaptation to tissue inflammation.

A second, larger proportion of eTreg cells express FOXP3+ RORγt+ Helios- Nrp1- and are classed as Th17-like Treg cells. RORγt Treg cells constitute 40-60% of total colonic Treg cells (96, 101) and are normally directed against microbiota, constraining intestinal immune responses (94, 102). Recently, RORγt expression in colonic Treg cells has been associated with maintenance of Foxp3 expression during colitis (103). Deletion of RORγt was found to lead to an upregulation of T-bet and IFN-γ, loss of Foxp3 expression, and severe intestinal inflammation in mice. T-bet deletion in RORγt knockout animals was found to restore Foxp3 expression and immunosuppressive function during inflammation. These results suggest that RORγt, by suppressing T-bet expression, promotes Foxp3 expression and thereby Treg cell function (103). Moreover, RORγt expression correlates with upregulation of immunosuppressive receptors including PD-1, ICOS, and CTLA4, the nucleotidases CD39 and CD73, and secretion of high amounts of anti-inflammatory cytokines including IL-10 and TGF-β. This transcriptional reprogramming enhances regulatory function to restrain inflammatory responses critical for controlling chronic inflammation (96, 101, 104).

Taken together, these findings illustrate a range of Treg cell phenotypes and functions in the intestinal mucosa, with remarkable flexibility to maintain tissue homeostasis. While transcriptional analysis of mice colonic Treg cells identified Gata3^+^Helios^+^ and RORγt^+^ Helios^−^ subpopulations, in humans this is less clear. GATA3^+^ Treg cells have been described in blood, however, they have not yet been identified in human intestine (97). RORγt^+^ Treg cells are present in comparable levels in biopsies from healthy colonic LP and patients with Crohn’s disease (96). Furthermore, RORγt^+^ Treg cells are elevated both in blood and tissues of IBD patients, and in the blood and tumors of CRC patients during different stages of the disease (105, 106).

## Tumor-Infiltrating Treg Cells and CRC

Since the observation of a population of immunosuppressive CD4+ T-cells in sarcoma tumor-bearing mice (107), a steadily increasing number of studies has demonstrated an important role for Treg cells regulating anti-tumor immunity (108–110). Treg cells can account for more than 50% of all CD4+ T cells in the TME of solid tumors including gastric (111), lung (112), breast (113), ovarian (114), cervical (115), melanomas (116) and hepatocellular (112) cancer. As already mentioned, high numbers of TI-Treg cells is often associated with poor prognosis and low survival rates (117, 118). In CRC, increased numbers of FOXP3+ T cells correlate with both, improved (30, 71, 117, 119–121) or worsened prognosis and overall survival (122). This discrepancy may be explained by the difficulties in defining the identity of suppressive FOXP3+ Treg cells at the tumor site. In line with this, analysis of TILs in human CRC identified two heterogeneous subpopulations of FOXP3+ T-cells associated with patient outcome (123). Better prognosis was associated with increased infiltration of non-suppressive FOXP3lo non-Treg cells (CD45RA- FOXP3lo). These were found to generate a strong pro-inflammatory environment through the secretion of inflammatory cytokines such as TGF-β, IL-12, and TNF-α. In contrast, increased numbers of suppressive CD45RA- FOXP3hi eTreg cells correlate with poorer outcomes and lower disease-free survival (123).

The percentage of Treg cells, as a proportion of CD4+ T cells, infiltrating the TME in CRC is significantly higher compared to healthy colon (124). While some studies support the concept that TI-Treg cells may originate from tTreg cells that migrate and/or expand in the TME, others support *in situ* generation of pTreg cells (28, 125–127). TI-Treg cells expressing Helios have also been identified suggesting their thymic origin in human CRC (128). Studies analyzing CD4+ TCR repertoires in murine solid tumor models have demonstrated largely distinct TCR repertoires in Foxp3+ and Foxp3− CD4+ T cells. This suggests that increased numbers of TI-Treg cells is due to the *in situ* proliferation of Treg cell clones in tumors, and not conversion of CD4+ T cells to Treg cells (129, 130). Consistent with these findings, additional studies of TCR repertoires support that TI-Treg cells have little overlap with conventional CD4+ T-cells in murine models of prostate cancer (131) or human breast cancer (128). In CRC patients, Teff and Treg cells have been shown to develop distinct TCR repertoires against tumor-associated antigens, leading to specific tumor-associated antigen responses. Treg cells have high specificity for a limited repertoire of tumor antigens and exert their immunosuppressive function against Teff cells to damp their responses. Since the repertoire of antigens that Teff and Treg cells can detect is different, this provides the potential for therapeutic approaches that identify peptides that can stimulate anti-tumor T-cell but not Treg cell responses (132). A recent study characterizing the TCR repertoires of TI-Treg cells in human metastatic gastrointestinal melanoma, and ovarian cancers showed a significant overlap with circulating Treg cells but not with conventional CD4+ T cells found in either tumor or blood (133).

TI-Treg cells generally exhibit CpG hypomethylation patterns required for the induction of *Foxp3* expression and generation of stable, functional Treg cells (78, 85, 134). In colonic RORγt+ Treg cells, the CNS2 region, also referred to as the major Treg-specific demethylated region (TSDR), is significantly demethylated (101, 135). In CRC tumors, TSDR demethylation has been associated with STAT5 and TET2, and the expression of both is upregulated in CRC tumor-infiltrating CD4^+^ T cells. Here, STAT5 and TET2 coordinately bind the FOXP3-TSDR, promoting DNA hypomethylation and *FOXP3* expression (136). Analysis of the *FOXP3*-TSDR demethylation rates (TSDR-DMRs) in Treg cells from CRC tumors and adjacent healthy colon samples demonstrated higher TSDR-DMRs demethylation in TI-Treg cells (137). In another study utilizing 130 paired samples of CRC tumor tissue and adjacent healthy colonic mucosa samples, significantly higher TSDR-DMRs and increased *FOXP3* mRNA and protein expression were also observed in TI-Treg cells (138). These observations provide an explanation for increased FOXP3 expression in CRC tumor-infiltrating Treg cells compared to healthy colon tissue.

**Figure 1:**
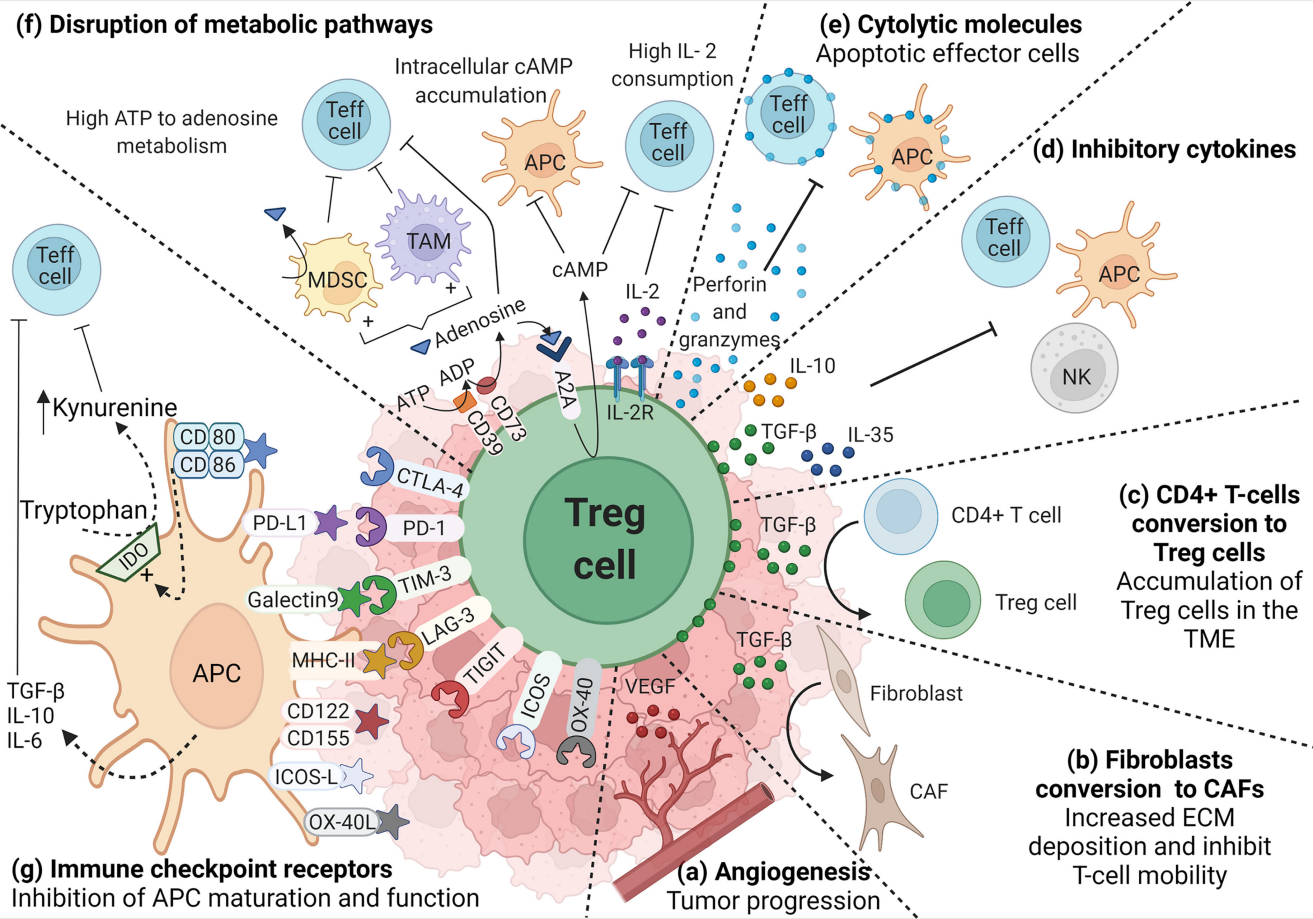
Role of TI-Treg cells in the TME. TI-Treg cells produce **(A)** VEGF to promote dysregulated angiogenesis associated with tumor progression and TGF-β that, **(B)** promote the conversion of fibroblast to cancer-associated fibroblasts (CAFs), and **(C)** the conversion of CD4+ T-cells to Treg cells promoting their accumulation in the TME. TI-Treg cells also regulate anti-tumor immune responses by producing **(D)** inhibitory cytokines, such as IL-10, TGF-β and IL-35, inhibiting Teff cells, NKs and APCs, the last two are also inhibited through membrane-bound TGF-β, and **(E)** cytotoxic molecules such as granzymes and perforin that can directly kill Teff cells and APCs. TI-Treg cells also **(F)** disrupt Teff cell intracellular metabolism impairing their function by depleting IL-2 in the TME. They express CD39 and CD73 ectonucleotidases that covert ATP and ADP into adenosine, which can engage adenosine receptor A2A on the surface of Teff cells, increasing intracellular cAMP and disrupting their metabolism and function. cAMP also binds to APCs and macrophages inducing tolerogenic myeloid-derived suppressor cells (MDSCs) and tumor-associated macrophages (TAMs) that further impact Teff cells. Furthermore, adenosine can also bind A2A in Treg cells promoting the intracellular accumulation of cAMP that can be transferred through gap junctions to Teff cells interfering with their metabolism. Among different molecules that participate in the suppression process, Treg cells highly express **(G)** immune checkpoint receptors that bind their corresponding ligand in APCs and differently regulate their function (increasing the release of inhibitory cytokines or the upregulation of IDO) promoting an immunosuppressive TME.

The CRC TME can also promote the conversion of CD4+ T-cells to TI-Treg cells through a variety of ways, for example increasing the availability of TGF-β (**Figure 1C**) (139). Other mechanisms associated with the differentiation of CD4+ T cells to Treg cells include the increased expression of indoleamine 2,3-dioxygenase (IDO) in mouse and human CRC TME. IDO converts tryptophan to kynurenine, which can bind the aromatic hydrocarbon receptor (AhR) in CD4+ T cells thereby promoting Foxp3 expression (140, 141). Treg cells localize to the CRC TME through their expression of specific chemokine receptors. These include: CC chemokine receptor 4 (CCR4), attracted to inflammatory loci by the ligands CCL22 or CCL17 produced by CRC tumor cells or macrophages (142); CCR5, highly expressed in TI-Treg cells and associated with Treg cell recruitment towards the ligands CCL3, and CCL4 expressed by CRC tumor cells (34, 143); CCR6, involved in migration to the TME in response to CCL20 produced by tumor-associated macrophages (144, 145); CCR8, exclusively expressed by TI-Treg cells (146) and CCL1, expressed by tumor-infiltrating myeloid cells (128). Taken together, multiple mechanisms are involved in the increased numbers of Treg cells in the context of CRC. This can involve accumulation in the TME by chemotaxis and clonal expansion by recognition of tumor-associated antigens.

TI-Treg cells are more proliferative and have a more activated immunosuppressive phenotype when compared to Treg cells from healthy tissue or peripheral blood (147). Helios, associated with Treg cell thymic origin, is highly expressed in CRC Foxp3hi TI-Treg cells which also co-express activation markers such as ICOS and OX-40, and inhibitory molecules such as PD-1, CTLA-4, TIM-3 (**Figure 1G**), and CD39 that mediate their highly suppressive phenotype (**Figure 1F**) (148). CD39 is an ectonucleotidase that catalyzes the generation of adenosine from ATP, negatively regulating immune responses (149). A recent study comparing CRC tumors and healthy colon tissues identified three clusters of Treg cells differentially expressing CD39. The subgroups with the highest and intermediate expression of CD39 were found in the tumor, while the subgroup with the lowest expression was found in healthy colon tissue. Higher expression of CD39 correlated with poorer outcome (150).

In the context of CRC, the most significantly enriched TI-Treg cell population has a Th17-like profile. IL-17^+^ TI-Treg cells, originated from memory CCR6+ T cells, cTreg cells, or the transdifferentiation of Th17-to-Treg cells, accumulate in colitis inflamed tissue and are associated with CRC progression (151–153). These cells maintain transcriptional and epigenetic signatures of both Th17 cells (*Folr4, GARP, Itgb8, Pglyrp1, Il1rl1* and *Itgae*) and Treg cells (*Foxp3*, *Tigit* and *Icos*) (135, 153). IL-17^+^ TI-Treg cells also acquire RORγt expression contributing to local inflammation by producing IL-17, IL-2, IL-6, TNF and IFN-γ, in combination with the capacity to inhibit T cell immunity, thereby driving initiation of tumorigenesis (105, 106, 153–156). RORγt expression is increased and promoted by Wnt signaling. In healthy colonic Treg cells, TCF-1 and FOXP3 co-bind to the enhancer regions of pro-inflammatory genes such as *Rorc* (RORγt), *Il17a* (IL-17) and *Ifng* (IFNγ), repressing their expression. However, in CRC RORγt+ TI-Treg cells, sustained Wnt and/or TCR signaling leads to nuclear translocation of β-catenin where it binds to TCF-1 facilitating the transcription of pro-inflammatory genes. Increased numbers of pathogenic β-catenin(hi) RORγt+ TI-Treg cells correlates with the progression from IBD to CRC in humans and in murine colitis-associated dysplasia models (105, 157). CRC-associated Treg cells have decreased TCF-1 expression and increased Th17 expression signatures compared to normal tissues. Reduced TCF-1 activity in Treg cells increases their immunosuppressive function against T cell proliferation, but at the same time promotes their pro-inflammatory activity, promoting tumor growth in polyp-prone mice (158). Conversely, the specific ablation of RORγt in Foxp3+ Treg cells of polyp-prone mice improves anti-tumor responses and reduces polyposis (106, 159).

Epigenetic modifications at the promoter regions of TI-Treg cell genes promote immunosuppressive function and support tumor immune evasion. Expression levels of immune checkpoint proteins such as PD-1, CTLA-4, TIM-3 and TIGIT, and checkpoint ligands including PD-L1 and galectin-9 are higher in peripheral blood or tumor tissue for CRC patients, compared with blood or colon tissue from healthy donors (160, 161). Increased DNA hypomethylation at the promoters of CTLA-4 and TIGIT in TI-Treg cells, compared to healthy colon tissue, correlates with increased expression of TETs. Moreover, the distribution of the repressive histone modifications H3K27me3 in CTLA-4 and TIM-3, and H3K9me3 in PD-1, TIM-3 and TIGIT, is lower in TI-Treg cells correlating with higher expression of these immune checkpoint receptors (160). RORγt^+^ Treg cells retain a suppressive phenotype through hypomethylation of Treg-specific signature genes including Foxp3, Ctla-4, Gitr, Eos, and Helios (135, 151, 152, 154). Their immunosuppressive activity in the TME is thought to occur through the release of factors such as TGF-β (**Figure 1D**), which is upregulated in around 80% of the RORγt^+^ TI-Treg cells. A smaller proportion also expresses IL-10 (**Figure 1D**), and they can also release IL-6, IL-4, IFN-γ, and TNF-α (106, 154). RORγt^+^ TI-Treg cells can also co-express the BLIMP1 transcription factor identified as the primary regulator of IL-10 expression in the colon, suppressing inflammation-driven CRC (162). BLIMP1+ TI-Treg cells have been associated with longer disease-free survival in CRC patients (163). In summary, RORγt has an important role in the balance between suppressive Treg cells and pathogenic RORγt^+^ Treg cells that can contribute to an inflammatory and immunosuppressive environment that can promote both CRC tumor development and immune evasion.

The majority of TI-Treg cells express co-inhibitory receptors such as LAG-3 and TIM-3 that can modulate T cell responses (**Figure 1G**) (164). LAG-3+ Treg cells have increased IL-10 and TGF-β production and are enriched both in the TME and peripheral blood of CRC patients (165). TIM-3+ Treg cells have been associated with multiple cancers including CRC and have a role in regulating immune responses by driving T cell inhibition or exhaustion. They express CD39/CD73 (**Figure 1F**), IL-10, and TGF-β (**Figure 1D**) and upregulate the expression of checkpoint receptors (**Figure 1G**), such as CTLA-4, PD-1, and LAG-3 (166–170). CRC tumors implanted in TIM-3 deficient mice have a reduced infiltration of Treg cells, reduced CD8+ Teff cell exhaustion, and slower tumor growth (170). LAG-3+TIM-3+ TI-Treg cells are also enriched in CRC tumors compared to healthy colon, with increased TGF-β and IL-10 release, and upregulation of CTLA-4 expression compared to LAG-3−TIM-3− TI-Treg cells. In addition, LAG-3+TIM-3+ TI-Treg cells can reduce macrophage expression of MHC class II, CD80/CD86, and TNF-α and increase IL-10 secretion thereby supporting immunosuppression (171).

Additional CRC-related Treg cell subsets have been described, although less is known about their functional relevance. TI-Treg cells producing IL-35 have been identified in CRC patients (**Figure 1D)**; however, their origin, phenotype, and function remain to be defined. IL-35 has immunosuppressive activity both *in vitro* and *in vivo*, inhibiting Teff cell proliferation and inducing conversion to IL-35-producing Treg cells (172–174). Studies with CRC patients and mouse models found increased TI-Treg cell numbers correlated with high levels of IL-35 expression in serum and TME (173–176). Latency-associated peptide (LAP) non-covalently binds to TGF-β and forms a latent TGF-β complex inhibiting the interaction with its cognate receptors on immune cells (177). LAP+ TI-Treg are functionally more immunosuppressive than LAP- TI-Tregs, and their numbers increase with CRC progression and the metastatic stage (178, 179). A study of 42 CRC patients concluded that LAP+ TI-Treg cells were increased in the peripheral blood and tumor tissue of patients compared to healthy colon controls. These Treg cells upregulated effector molecules including tumor necrosis factor receptor II, granzyme B, perforin, Ki67, and CCR5 that further support their immunosuppressive function. LAP+ TI-Treg cells can direct cell-mediated cytotoxicity through the expression of perforin and granzyme B, thereby also suppressing Teff cells (**Figure 1E**) (178).

Taken together, Treg cell plasticity and heterogeneity provide a broad range of mechanisms to temper anti-tumor immune responses and promote immune evasion. It is therefore important to accurately characterize Treg cell populations to improve the diagnosis and outcome of CRC patients and more specifically target TI-Treg cells.

## The TME Promotes Treg Cell Metabolic Reprogramming

The reprograming of energy metabolism is one of the hallmarks of cancer whereby tumors can influence the immunosuppressive TME. In CRC, this already takes place at the colon adenoma stage (180) where CRC initiation, proliferation, invasion, and metastasis are closely associated with the metabolic crosstalk between tumor cells, the TME, and the microbiota (181). CRC tumor cells reprogram their metabolism in order to fulfill their excessive energy and nutrient needs, relying on aerobic glycolysis, glutaminolysis, and fatty acid synthesis. As a result of the rapid proliferation of tumor cells and the immaturity of tumor vasculature, the TME is characterized by hypoxia, acidity, and nutrient deficiency including glucose, glutamine, and tryptophan, with enrichment of lactate and kynurenine (182, 183). These metabolic changes hamper effective Teff cell activation and proliferation, which themselves rely on aerobic glycolysis. In contrast, this harsh metabolic environment promotes the recruitment and differentiation of Treg cells (184). FOXP3 is a central regulator of TI-Treg cell metabolic adaptation, driving a distinct metabolic profile compared to Teff cells. TI-Treg cells are less dependent on glycolysis, increasing FA-oxidation (FAO) and oxidative phosphorylation (OXOPHOS) to support their differentiation and function (185, 186). In CRC, as in other solid tumors, TI-Treg cells utilize these alternative metabolic pathways to produce energy, proliferate and perform their immunosuppressive functions (187–189).

**Figure 2:**
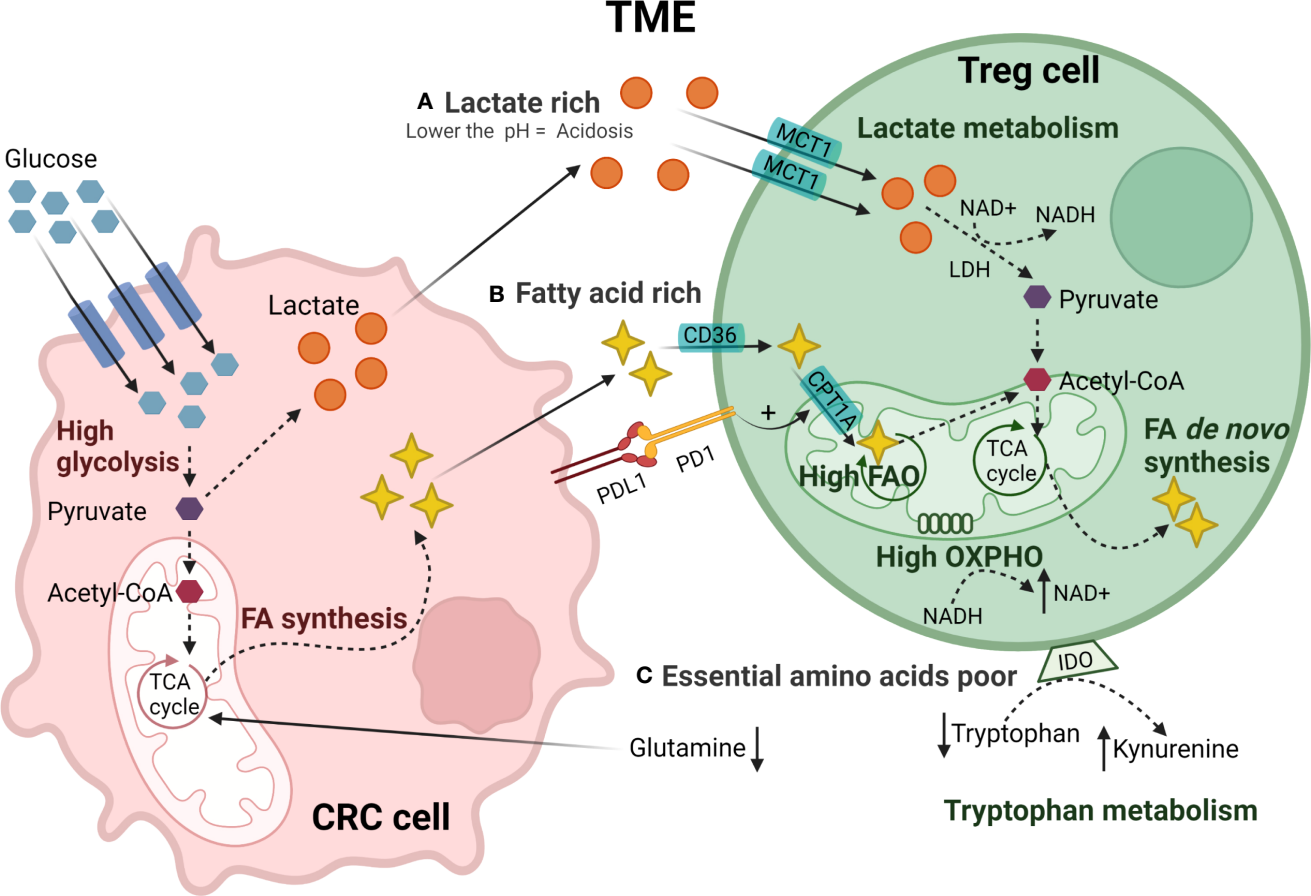
Metabolic reprogramming of TI-Treg cells in CRC TME. CRC tumor cells have a high glucose uptake generating pyruvate through glycolysis. Pyruvate is converted to acetyl-CoA or lactate that is secreted creating **(A)** a lactate-rich TME. TI-Treg cells adapt to these metabolic stresses present in the TME. TI-Treg cells can take up lactate through the MCT1 transporter that is subsequently converted to pyruvate by LDH and further to acetyl-CoA. Moreover, CRC tumor cells fuel the tri-carboxylic acid (TCA) cycle in the mitochondria for FA synthesis creating a **(B)** fatty acid-rich TME. TI-Treg cells can take up FA from the TME through the fatty acid transporter CD36. The CPTR1A transporter drives FA uptake into the mitochondria where it is oxidized by fatty acid oxidation (FAO) to acetyl-CoA. Acetyl-CoA fuels the TCA cycle in the mitochondria for *de novo* fatty acid synthesis that can be accumulated intracellularly or exported to the TME. The TME has **(C)** low availability of essential amino acids, particularly glutamine, that is consumed by tumor cells, and tryptophan, that is catabolized to kynurenine by IDO highly expressed by Treg cells.

Accumulation of lactate (**Figure 2A**), the end-product of tumor cell glycolysis, affects the TME through multiple mechanisms. Treg cells can metabolically adapt to increased lactate in the TME, utilizing it as a carbon source for intracellular metabolism. TI-Treg cells upregulate pathways related to the metabolism of lactate such as lactate dehydrogenase (LDH), and the lactate transporter MCT1 (SLC16A1) (190, 191). FOXP3 directly alters T cell metabolism to maintain Treg cell suppressive function in lactate-rich environments (185). By binding to the *MYC* promoter, FOXP3 can suppress c-myc expression, a transcription factor regulating glycolytic gene expression, thereby suppressing glycolysis. FOXP3 also influences LDH activity, promoting the conversion of lactate to pyruvate and increasing oxidative phosphorylation (OXPHOS), and the NAD : NADH ratio. These adaptations permit Treg cells to differentiate and work effectively under conditions of low-glucose and high lactate as found in the TME (185).

Besides lactate, TI-Treg cells also rely on fatty acids (**Figure 2B**), utilizing lipid metabolism to meet both their energy demands and immunosuppressive function through FAO-driven OXPHOS (185, 186). Fatty acids stimulate the AMPK pathway, inhibiting mTOR signaling and promoting mitochondrial FAO (186). TI-Treg cells also upregulate SREBP transcription factors in order to adapt to the TME. SREBPs regulate the expression of genes required for *de novo* synthesis of lipids by fatty acid synthase (FAS), promoting the functional maturation of TI-Treg cells. SREBP-deficient Treg cells show decreased expression of PD-1 and have been associated with an effective anti-tumor immune response and reduced tumor growth (192). PD-1 signaling in TI-Treg cells can also promote FAO by upregulating palmitoyltransferase-1A (CPT1A), an importer of fatty acids to the mitochondria (193). A study in CRC, among other solid tumors, showed that high levels of fatty acids in the TME are an essential energy source for TI-Treg cells (188). TI-Treg cells also increase intracellular lipid accumulation through increases in fatty acid synthesis (188). In a CRC murine model, TI-Treg cells were found to upregulate CD36, an FA-importer, increasing FA uptake and FAO (194). This metabolic flexibility allows TI-Treg cells to thrive and supports their suppressive activity in the TME under conditions of high concentrations of tumor-derived lipids (195).

Under homeostatic conditions, amino acids regulate the activation of mTORC1 which is important for the functional fitness of eTreg cells (196, 197). However, the TME generally has low availability of many amino acids (**Figure 2C**), particularly glutamine. Reduced levels of glutamine, with the subsequent reduction of glutamine catabolism, stimulates the conversion of CD4+ T cells to Treg cells during Th1 polarization *in vitro* (198, 199). In addition, the TME often has high levels of amino acid-degrading enzymes such as IDO. IDO is expressed by both tumor cells and Treg cells and, as already mentioned, catabolizes tryptophan to kynurenine, binding to AhR in Teff cells and promoting the Treg cell differentiation (200–205). High IDO expression by CRC cells and tryptophan depletion in the TME has been associated with tumor immune evasion and increased Treg infiltration in CRC (206).

Other metabolites abundant in the TME include retinoic acid (RA) and adenosine. RA can act as a cofactor to induce Foxp3 expression and promote the conversion of CD4+ T cells into Treg cells while inhibiting the generation of Th17 cells. In the intestine, CD103+ DCs can generate RA from vitamin A and drive Treg cells differentiation in the presence of TGF-β (207, 208). A colitis-associated CRC murine model treated with RA showed an increased frequency of TI-Treg cells in the tumor and reduced inflammation (209). Adenosine levels are increased in response to hypoxia and chronic inflammation, both characteristics of CRC. Treg cells can produce adenosine by CD39/CD73 action but can also respond to it by engaging the adenosine receptors (A_2A_ AR) (**Figure 1F**). A_2A_ engagement promotes the intracellular accumulation of cAMP and, subsequently, the activation of the cAMP response element-binding protein (CREB) that drives the expression of anti-inflammatory cytokines, including IL-10 and TGF-β (210, 211).

Despite the metabolic challenges present in the TME such as low glucose and amino acid availability, high lactate and lipid concentration, acidity, and hypoxia, TI-Treg cells show a remarkable ability to engage unique metabolic reprogramming, compared to other Teff cells, to support their bioenergetic and functional needs. Understanding the TI-Treg cell metabolic reprogramming potentially provides novel therapeutic approaches for targeting in the control CRC pathogenesis.

## Therapeutic Approaches to Target Treg Cells in CRC

Modulating TI-Treg cell function offers an important strategy to improve therapeutic responses for a wide variety of tumors. A variety of approaches are currently under development including Treg cell depletion, suppressing their activity, impeding their recruitment, and preventing their differentiation within the TME. The challenge presented is to specifically target TI-Treg cells without affecting other Treg populations, thereby diminishing the risk of developing unwanted autoimmune responses. In the past decade, the efficacy of checkpoint inhibitors to block immune checkpoint receptors or their ligands has greatly benefited the survival of patients with solid tumors (212–216). As discussed earlier, CRC TI-Treg cells express immune checkpoint receptors including PD-1, CTLA-4, TIM-3, and the NTPDase CD39 that regulate their immunosuppressive phenotype and support tumor cells in escaping immune surveillance (**Figure 1G**) (148, 217). A variety of humanized monoclonal antibodies developed against these receptors have been evaluated in CRC patients in order to alter TI-Treg cells numbers and functionality (**Table 2**).

**Table 2:** CRC clinical trials potentially targeting Treg cell populations.

Clinical trial	Antibody target	*n* CRC patients and tumor type	Response to treatment	PFS	OS	Comments and references
NCT01876511 (Phase II)	**anti-PD-1** pembrolizumab (MK-3475, SCH 900475)	10 metastatic MSI CRC patients 11 metastatic MSS CRC patients	40% MSI ORR 0% MSS ORR	78% MSI 11% MSS		This findings drove FDA-approval for the treatment of unresectable, metastatic MSI-H and dMMR (218)
NCT00441337 (Phase I)	**anti-PD-1** nivolumab (BMS-936558, MDX-1106, ONO-4538)	14 metastatic MSI or MSS CRC patients	1 MSI CR ≥ 21 months			Followed up in: NCT02060188 (phase II) (219)
NCT02060188 (Phase II)	**anti-PD-1** nivolumab	74 refractory metastatic dMMR/MSI-H CRC patients	23 patients PR 31% ORR 69% DCR ≥ 12 weeks	50% ≥ 12 months	73% ≥ 12 months	This findings drove FDA-approval for the treatment of refractory MSI-H/dMMR CRC (212)
	**anti-PD-1** nivolumab in combination with **anti-CTLA-4** ipilimumab (IgG1 isotype)	119 refractory metastatic dMMR/MSI-H CRC patients	55% ORR 80% DCR ≥ 12 weeks	71% ≥ 12 months	85% ≥ 12 months	Combination therapy improves therapeutic efficacy for dMMR/ MSI-H CRC (220)
NCT00313794 (Phase II)	**anti-CTLA-4** tremelimumab (CP-675,206 or ticilimumab)	47 refractory metastatic CRC patients Subtype not determined	45 response-evaluable patients	2% 15 months before relapse	45% ≥ 6 months	No clinically meaningful but interesting for combinational approaches (221)
NCT02870920 (Phase II)	**anti-CTLA-4** tremelimumab and **anti-PD-L1** durvalumab	180 pre-treated-refractory MSS or proficient MMR CRC patients		1.8 months	6.6 months	Combination therapy improves the OS and quality of life of patients with advanced refractory CRC but not dMMR CRC (222)
NCT03101475 (Phase II)	**anti-CTLA-4** tremelimumab and **anti-PD-L1** durvalumab	Currently, 22 metastatic CRC patients				Ongoing clinical trial
NCT02794571 (Phase I)	**anti-TIGIT** (MTIG7192A) as monotherapy or in combination with **anti-PD-L1** atezolizumab	Recruiting advanced incurable tumors, including CRC patients				Ongoing clinical trial
NCT01968109 (Phase I/IIa)	**anti-LAG-3** relatlimab (BMS-986016) as monotherapy or in combination with **anti-PD1** nivolumab	Recruiting advanced solid tumors including CRC patients				Ongoing clinical trial
NCT03156114 (Phase I)	**anti-LAG-3** miptenalimab (BI 754111) as monotherapy or in combination with **anti- PD-1** ezabenlimab (BI 75409111)	Recruiting advanced solid tumors including metastatic CRC patients				Ongoing clinical trial
NCT02608268 (Phase I/II)	**anti-TIM-3** (MBG453) with **anti-PD-1** spartalizumab (PDR001)	6 metastatic CRC patients	2 partially responded			Ongoing clinical trial (223)
NCT02817633 (Phase I)	**anti-TIM-3** (TSR-022) as monotherapy or in combination with **anti- PD-1** dostarlimab (TSR-042)	Recruiting advanced solid tumors including CRC patients				Ongoing clinical trial
NCT02705105 (Phase I/II)	**anti-CCR4** mogamulizumab as monotherapy or in combination with **anti- PD-1** nivolumab	29 MSS CRC patients	0 ORR with monotherapy 1 ORR with combination therapy			No enhanced efficacy of the combination therapy compared to monotherapy with nivolumab (224)

PFS, progression-free survival; OS, overall survival; MSI, microsatellite instability tumors; MSS, microsatellite stable tumors; dMMR, different mismatch-repair; ORR, overall response rate; PR, Partial response; CR, Complete response; DCR, disease control rate; BSC, best supportive care.

PD-1 is highly expressed in TI-Treg cells and plays a role in their homeostasis and function. PD-1 signaling also promotes FAO to support their bioenergetics needs in the TME (193). PD-1-expressing Treg cells are considered a critical element in tumor immune evasion and progression of CRC (225). PD-1, expressed on tumor cells and Treg cells, can bind its ligand PD-L1 on Teff cells activating inhibitory signals that interfere with the TCR signal transduction thereby blocking anti-tumor immune responses (226, 227). Humanized monoclonal anti-PD-1 antibodies (mAbs) block interaction with PD-L1 and can thereby benefit the anti-tumor immune response (217, 228). Recently, it has been demonstrated that the balance of PD-1 expressing CD8+ Teff cells and eTreg cells in the TME is relevant in predicting the efficacy of anti-PD-1 mAbs (229), although how PD-1 signaling promotes Treg cell suppressive function is unclear. A study in CRC patients showed that anti-PD-1 mAb therapy has a better outcome for patients with high numbers of PD-1+ CD8+ T cells compared to non-responders that have higher numbers of eTreg cells. In line with these previous results, a CRC murine model demonstrated the capacity of anti-PD-1 mAbs to restore the effector function of PD-1+ CD8+ T cells and promote tumor regression (230). Clinical trials have investigated the possibility of using humanized anti-PD-1 monoclonal antibodies for the treatment of CRC patients (**Table 2**). Promising clinical results with the first mAb against PD-1, pembrolizumab (MK-3475, SCH 900475) and, later, nivolumab (BMS-936558, MDX-1106, ONO-4538) in CRC patients lead the U.S. Food and Drug Administration (FDA) to approve them for the treatment of refractory metastatic CRC that still progressed after prior treatment with chemotherapeutics (212, 231). The diverse outcomes observed in CRC patients treated with different anti-PD-1 mAbs can be explained according to the subtype of CRC tumor. Unfortunately, few studies have reported responses regarding the targeting of TI-Treg cells by these antibodies in CRC patients. However, based on current knowledge it is likely that anti-PD-1 mAbs target Treg cells and in this way support activation of an anti-tumor immune response.

Treg cells express high levels of cytotoxic T lymphocyte antigen 4 (CTLA-4) in a FOXP3-dependent manner. CTLA-4 binds and inhibits B7 molecules (CD80 and CD86) on the surface of APCs such as DCs with higher affinity than their CD28 co-stimulatory signal. This results in the induction of immunological tolerance (230). Murine CRC models have shown that anti-CTLA4 mAbs induce a potent immune response, rejection of the tumor, and significantly prolonged survival (232). Humanized anti-CTLA-4 mAbs such as ipilimumab (IgG1 isotype) and tremelimumab (IgG2 isotype) have been tested in multiple clinical trials with solid tumors (233–235). The anti-tumor mechanism of these anti-CTLA-4 mAb was first attributed to preventing interaction of CTLA-4 with its ligand B7 allowing APCs to present antigens and activate anti-tumor T cells response. Recently, it has been shown that anti-CTLA-4 mAbs also deplete TI-Treg cells, but not pTreg cells in secondary lymphoid organs. This selective Treg cell depletion depends on their ability to activate Fc receptors on tumor-associated macrophages or NK cells that can subsequently phagocytose or kill Treg cells (234, 236–241). The use of anti-CTLA-4 (IgG2a isotype) in two murine subcutaneous CRC tumor models demonstrated successful reduction of TI-Treg cells together with the expansion of CD8+ Teff cells promoting anti-tumor activity (238). The use of anti-CTLA-4 mAb in clinical trials for patients with metastatic CRC, overall, has shown improved therapeutic efficacy in combination with anti-PD-1 mAbs compared to anti-CTLA-4 mAb monotherapy (**Table 2**) (212, 220, 222). Currently, the dual combination of checkpoint inhibitors is being evaluated in various phase II clinical trials for metastatic CRC patients, for example, durvalumab, an anti-PD-L1 mAb, and tremelimumab, an anti-CTLA-4 mAb (NCT03101475). There have been no specific reports on anti-CTLA-4 mAb targeting TI-Treg cells in these CRC patients, but again it is likely that TI-Treg cell function and numbers are impacted. Anti-CTLA4 mAbs have also been shown to have a T-cell intrinsic mechanism of action that enhances the proliferation of Teff and Treg cells in response to self-antigens as shown in human and mouse models (130, 242, 243).

Novel immune checkpoint targets such as TIGIT, LAG-3 and TIM-3 are currently under pre-clinical investigation and are being evaluated for their safety profiles in phase I trials (244, 245). In CRC, these receptors are highly expressed on TI-Treg cells compared to healthy colon tissue (150). TIGIT is a co-inhibitory molecule, a member of the CD28 family, expressed preferentially in T cells and NK cells. TIGIT competes with the co-stimulatory receptor CD226 in T-cells to bind CD155 on APCs which become tolerogenic, cannot activate T-cells, and release IL-10 inhibiting of Teff cell anti-tumor responses (246–248). A phase I clinical trial (NCT02794571; Genentech) utilizing humanized anti-TIGIT mAb (MTIG7192A) as monotherapy or in combination with anti-PD-L1 mAb (atezolizumab) is ongoing in advanced or metastatic tumors including CRC (**Table 2**). LAG-3 is expressed on activated CD4+ and CD8+ T cells (249), Tregs (250), a subpopulation of natural killer (NK) cells (251), B cells (252), and plasmacytoid dendritic cells (pDCs) (253). The LAG-3 co-inhibitory receptor can bind stable peptide-MHC-II complexes impairing DC function, maturation, and proliferation. LAG-3 can also induce the production of IDO, impairing Teff cell and DC proliferation, but promoting eTreg cell differentiation (165, 230, 254, 255). In CRC patients, LAG-3+ Treg cells have been identified in both TILs and in peripheral blood (165). Currently, there are two ongoing clinical trials that aim to investigate the efficacy of anti-LAG-3 mAb alone or in combination with anti-PD-L1 mAb in patients with advanced solid tumors including CRC (**Table 2**). The rationale behind this combinational treatment is to synergistically restore T cell activation and enhance antitumor immunity.

TIM-3 is another immune checkpoint molecule expressed on innate immune cells such as DCs and NK cells (256). pTreg cells do not normally express TIM-3, however, TIM-3+ TI-Treg cells have been identified in the TME where they exert an inhibitory role against Teff cell responses mainly by driving their exhaustion (166–168, 257). This suggests a therapeutic advantage over other checkpoint receptors, such as CTLA-4 and PD-1, due to expression predominantly on TI-Treg cells. For CRC patients, the combination therapy anti-TIM-3 mAb and anti-PD-1 mAb has already been tested showing partial response (**Table 2**) (223). An ongoing Phase 1 clinical trial using a humanized anti-TIM-3 mAb (TSR-022) aims to evaluate first the dosage and, then, the anti-tumor capacity of the antibody in advanced solid tumors (NCT02817633). For CRC patients, anti-TIM-3 was administrated as monotherapy or in combination with an anti-PD-1 mAb, dostarlimab (TSR-042) (**Table 2**).

As already discussed, TI-Treg cells express high levels of CD39/CD73 ectonucleotidases that convert ATP to adenosine, a potent suppressor of tumor immunity (**Figure 1** **f**) (258). CD39+ TI-Treg cells have been found to play an important role during CRC tumor growth (150) and inhibition of CD39 enzymatic activity has been evaluated in a CRC hepatic-metastatic murine model. Here, CD39+ Treg cells modulate NK reactivity against the tumor, and targeting CD39 was found to both inhibit Treg cell activity *in vitro* and reduce tumor growth *in vivo* (259). Adoptive reconstitution of either wild-type or CD73-deficient Treg cells has been performed in Treg cell-depleted mice implanted with CRC-tumors. Only mice reconstituted with wild-type Treg cells effectively support tumor growth, suggesting a possible therapeutic benefit of targeting CD73-Treg cells (260).

CCR8 expression can discriminate between Treg cells infiltrating CRC and other solid tumors, from those found in secondary lymphoid organs (261). In solid tumors, CCR8 expressing cells migrate in response to CCL1 ligand secreted by cancer-associated fibroblasts (CAFs), M2-polarized tumor-associated macrophages and Treg cells (262–264). Murine tumor models including colorectal adenocarcinoma have been used to evaluate the efficacy of targeting CCR8+ Treg cells in the TME. Administration of monoclonal antibodies targeting either the receptor or ligand reduces the number of TI-Treg cells without affecting the pTreg cells, reinforcing the anti-tumor immune response (265). CCR4+ TI-Treg cells also contribute to the CRCTME, and CCR4 expression is increased in TI-Treg cells compared to those of healthy colon tissue (266). Mogamulizumab is an anti-CCR4 mAb that has been used in clinical trials for the treatment of solid tumors with the aim of depleting TI-Treg cells. It has been evaluated as monotherapy (NCT01929486) or in combination with other targeted therapies such as anti-PD-1 mAb (nivolumab) (NCT02705105). Dose, safety, and efficacy for combination therapy has been assessed in 114 patients with locally advanced or metastatic solid tumors, including 29 MSS-CRC patients. The result showed no enhanced efficacy of the combination therapy compared to monotherapy with nivolumab. In the case of the CRC patients, only one out of the 29 showed ORR, similar to previously reported results of single-nivolumab treatment where none responded (224). Further evaluation of chemotaxis receptor-targeting therapeutics may provide more selective therapeutic strategies avoiding targeting pTreg cells.

Other broader strategies effective in Treg cell depletion include drugs such as cyclophosphamide (CY). The administration of low-dose CY, a chemotherapeutic agent used to treat cancer, seems to predominantly affect Treg cells over other effector cells (Teff cells or NKs), as has been demonstrated in a variety of human cancers and animal models. Low-dose CY can lead to a reduction in Treg cell numbers, upregulation of pro-inflammatory cytokines, and boosts the innate antitumor immune response (267–270). In CRC murine and rat models, the efficacy of low-dose CY, inhibiting Treg cells, in combination treatment with mycobacterium bovis Bacillus Calmette–Guérin (BCG) has been evaluated. BCG has TLR-agonist activity and increases the capacity of DCs to mediate an efficient anti-tumor immune response. The combination therapy resulted in tumor regression with Treg cell depletion in the blood and lymphoid organs but also decreased the number of TI-Treg cells (271). The synergistic combination of IL-12 gene therapy in mice with subcutaneous CRC, pre-treated with low-dose CY, was found to induce a potent anti-tumor effect. Combined treatment reduced the number of MDSCs and increased the anti-tumor DC responses and the number of IFN-γ-secreting CD4+ T cells (272, 273). Depletion of Treg cells by low-dose CY also results in significant anti-tumor responses in advanced chemotherapy-resistant solid cancers (268). In a randomized clinical trial with inoperable metastatic CRC patients, low-dose CY treatment resulted in delayed tumor progression associated with an increase in IFNγ+ anti-tumor T-cell responses, and reduction of Treg cells, B cells, and NK cells (274, 275).

Refinement of combinational therapies targeting Treg cells while promoting anti-tumor immune responses is clearly a promising approach to treat CRC more effectively. However, it remains critical to consider CRC subtypes, and to specifically target TI-Treg cells, rather than systemically depleting all Treg or other Teff cells. These are issues that still require proper evaluation when considering new therapeutic strategies.

## Future Directions

CRC FOXP3+ TI-Treg cells are a heterogeneous immune population with specific gene signatures and distinct functional properties. The phenotype and function of Treg cells are both modulated and exploited by the TME to promote immune evasion, tumor progression, and resulting in poor prognosis. Knowledge concerning Treg cell adaptation to the TME is invaluable in designing therapeutic strategies to target TI-Treg cells. However, it remains hard to specifically target Treg cells in the context of the TME. This is compounded by the difficulty of studying Treg biology *in vivo* or developing valid models for evaluating immune cell-TME interactions *in vitro*. Immunotherapy approaches can stimulate anti-tumor immune responses of effector cells or inhibit immunosuppressive mechanisms. Refinement of combinational therapies targeting Treg cells together with promoting Teff cell anti-tumor immune responses provides promising strategies to treat CRC more effectively. However, Treg cell-directed immunotherapy for CRC patients has several limitations. To start with, the different CRC molecular subtypes likely have a critical role in determining the success of certain therapeutic approaches. Due to CRC heterogeneity, treatment stratification to the tumor subtype will likely be required. Moreover, a better understanding of the (sub)phenotypes and functional diversity of CRC TI-Treg cells is still needed. This is essential to avoid compromising immune hemostasis and thereby developing unwanted side effects. Utilizing bivalent antibody approaches may help relieve the problem of specifically targeting only TI-Treg cells, as long as appropriate tumor markers are also available (276). Importantly, the expression of therapeutic targets on both TI-Treg cells and Teff cells does not necessarily prevent their application. Differential expression levels and dynamic expression profiles can still provide preferential Treg cell depletion in the context of the TME. Besides antibody-based immunotherapy approaches, the application of small molecule inhibitors remains an avenue of active exploration to disable TI-Treg cells. For example, FOXP3 protein stability is maintained by both active acetylation and deubiquitination, and inhibiting these processes effectively disables Treg cell function (277, 278). As already discussed, Treg cells can thrive in the altered metabolic environment of the TME. Increasing our understanding of the metabolic interactions between infiltrating T cells and tumor cells may help to expose an Achille’s heel that can be used to inhibit TI-Treg cell function more specifically. Combining such approaches with checkpoint inhibitor therapy may help drive the differentiation of anti-tumor effector T cells, at the expense of Treg cells, which are then free to reduce tumor progression. Similarly, understanding the contribution of epigenetic mechanisms to the control of Treg cell suppressive capacity is also relevant. Particularly as several small-molecule epigenetic modifiers are FDA approved and utilized in the clinic (279). Hypomethylating agents, inhibitors of histone deacetylases, and bromodomain inhibitors are all relevant in this context. For example, both the bromodomain inhibitor JQ1, and the HDAC6 inhibitor ricolinostat were found to attenuate Treg cell suppressive function, facilitate immune-mediated tumor growth arrest, and lead to prolonged survival of mice with lung adenocarcinomas (280).

Taken together, these studies further highlight the potential of not only targeting Treg cell markers but also Treg cell biology, in developing novel approaches to effectively re-activate anti-tumor immunity in CRC.

## Author Contributions

SAR conceptualized, wrote, edited and approved the manuscript. PJC conceptualized, edited, and approved the manuscript. OK contributed to the revision and final approval of the manuscript.

## Funding

This work was supported by a Worldwide Cancer Research grant (Reference: 19-0371).

## Conflict of Interest

The authors declare that the research was conducted in the absence of any commercial or financial relationships that could be construed as a potential conflict of interest.

## Publisher’s Note

All claims expressed in this article are solely those of the authors and do not necessarily represent those of their affiliated organizations, or those of the publisher, the editors and the reviewers. Any product that may be evaluated in this article, or claim that may be made by its manufacturer, is not guaranteed or endorsed by the publisher.
